# Associations between steady-state pattern electroretinography and estimated retinal ganglion cell count in glaucoma suspects

**DOI:** 10.1007/s10633-022-09869-9

**Published:** 2022-04-04

**Authors:** Andrew Tirsi, Derek Orshan, Benny Wong, Vasiliki Gliagias, Joby Tsai, Stephen A. Obstbaum, Celso Tello

**Affiliations:** 1 Department of Ophthalmology, Manhattan Eye, Ear, and Throat Hospital, Northwell Health, New York, NY USA; 2 Donald and Barbara Zucker School of Medicine at Hofstra/Northwell, Hempstead, NY USA; 3 New York Institute of Technology College of Osteopathic Medicine, Glen Head, NY USA; 4 Diopsys Inc., Pine Brook, New York, NJ USA

**Keywords:** CSFI, PERG, Glaucoma, Mediation, Structure–function

## Abstract

**Purpose:**

To estimate retinal ganglion cell (RGC) count in glaucoma suspects (GS) and ascertain its relationships with steady-state pattern electroretinography (ssPERG) parameters.

**Methods:**

In this prospective cross-sectional study, 22 subjects (44 eyes) were recruited at the Manhattan Eye, Ear, and Throat Hospital. Subjects underwent complete eye examinations, optical coherence tomography, standard automated perimetry, and ssPERG testing. Eyes were divided into two groups based upon clinical data: healthy subjects and GS. RGC count was estimated using the combined structure–function index.

**Results:**

Estimated RGC count, average retinal nerve fiber layer thickness (ARNFLT), and average ganglion cell layer and inner plexiform layer thickness (GCIPLT) were reduced in GS eyes (*p* ≤ *0.001* for all parameters). Pearson correlations revealed that ssPERG magnitude and magnitudeD correlated with ARNFLT *(r* ≥ *0.53, p* < *0.001*), GCIPLT *(r* > *0.38, p* < *0.011)*, and estimated RGC count *(r* > *0.46, p* < *0.002).* Six mediation analyses revealed that estimated RGC count mediated the relationships among ssPERG parameters, ARNFLT, and GCIPLT.

**Conclusion:**

Steady-state PERG parameters demonstrated linear correlations with estimated RGC count. The associations among ssPERG parameters and structural measures were mediated by estimated RGC count.

**Supplementary Information:**

The online version contains supplementary material available at 10.1007/s10633-022-09869-9.

## Introduction

Glaucoma is a group of optic neuropathies characterized by retinal ganglion cell (RGC) dysfunction and death, which results in vision loss [1]. RGC losses are evident in early disease before the onset of visual symptoms [2], and the percentage of lost RGCs before vision loss occurs is highly variable [3, 4]. Recent investigations provided evidence that a percentage of dysfunctional, but viable RGCs were present in early disease [5, 6]. Electrophysiological studies assessing RGC function therefore hold potential for identifying individuals with glaucoma suspects (GS) at the highest risk of developing visual impairment [7, 8].

Pattern electroretinogram (PERG) is an electrophysiological test that utilizes a reversing checkerboard patterned stimulus to measure RGC function [9]. PERG has diagnostic potential in glaucoma [10, 11], and cross-sectional data consistently revealed PERG losses in GS [11–14]. PERG was shown capable of identifying RGC dysfunction years before average retinal nerve fiber layer (ARNFLT) losses were detected by OCT [15], and before vision loss was detectable with standard automated perimetry (SAP) [10]. PERG has excellent repeatability and reproducibility [16, 17], and recently, a user-friendly type of PERG, steady-state PERG (ssPERG), was developed, allowing for widespread utilization in clinical practice [18–20].

Histological studies in primates with experimentally induced glaucoma provided evidence that PERG losses were related to optic nerve damage [21]. In humans, ssPERG parameters decreased with age at a rate that paralleled RGC losses [22] and correlated with ganglion cell complex thickness [12] and ganglion cell and inner plexiform layer thickness (GCIPLT) [14, 23]. In a recent study, ssPERG parameters correlated with global and sectoral rim area after adjusting for disk area, which provided evidence that ssPERG had the ability to detect optic nerve morphological changes in early glaucoma [20]. Taken together, these data suggest that ssPERG parameters are directly related to RGC count. However, to the best of our knowledge, there have been no reports directly comparing these parameters in GS.

Empiric determination of RGC count in living subjects is not yet possible; however, statistical models for estimating RGC count have been described. One model is a combined structure–function index (CSFI), which derives estimates by combining SAP and OCT measurements [24]. RGC counts estimated with the CSFI closely resemble histological counts [25, 26], and recently, a longitudinal cohort study demonstrated that the CSFI performed better than individual SAP and OCT measurements in predicting disease progression in GS [27].

The purpose of this study was to estimate RCC count in GS and to evaluate the relationships among ssPERG parameters, estimated RGC count, and SD-OCT structural measures.

## Methods

In this prospective cross-sectional study, a total of 22 consecutive subjects (44 eyes) were recruited from the Manhattan Eye, Ear, and Throat Hospital ophthalmology practice and divided into healthy subjects and GS based on their clinical data. Participants underwent a complete ophthalmologic examination, including slit lamp biomicroscopy, Goldmann tonometry, standard automated perimetry (Humphrey Field Analyzer II, 24-2 SITA-Standard strategy), OCT (Carl Zeiss Meditec, Inc., Dublin, CA, USA), and ssPERG (Diopsys Inc., Pine Brook, NJ, USA).

Participants were 20–80 years old and had a best corrected visual acuity better or equal to 20/40 as measured by Snellen visual acuity testing at the time of enrollment. All participants had a suspicious glaucomatous optic nerve head appearance (increased cup-to-disk ratio > 0.4, or neuroretinal rim thinning, notching or excavation) and a documented and repeatable normal HFA 24-2 at the baseline visit. All participants were not on intraocular pressure lowering treatment at the time of enrollment. All individuals with prior intraocular or posterior segment intraocular surgery, ocular trauma, or ocular or systemic conditions that may affect optic nerve head structure and/or function, except for uncomplicated cataract extraction with posterior chamber intraocular lens implant and no escape of vitreous to the anterior chamber performed less than a year before enrollment, were excluded from this study. Two GS subjects and two age-matched healthy controls under the age of 40 were enrolled in this study. Glaucoma suspect subjects had a strong family history of open-angle glaucoma and suspicious optic nerve head findings.

### Spectral-domain optical coherence tomography

Average retinal nerve fiber layer thicknesses (ARNFLT) were measured using the Optic Disc Cube protocol of a Cirrus spectral-domain optical coherence tomography (SD-OCT) version 6.0 (Cirrus HD-OCT; Carl Zeiss Meditec, Inc., Dublin, CA). The protocol scans a 6 × 6 mm area centered around the optic nerve head, collecting 200 × 200 axial scans containing 40,000 points. ARNFLT is measured segmentally in quadrants and clock-hour sectors within a 3.46 mm region of interest centered around the fovea. Average and minimum GCIPLT were measured using the ganglion cell analysis provided by Cirrus SD-OCT version 6.0, which uses a 4.8 × 4.0 mm oval-shaped region of interest centered around the fovea. Similar to the Optic Disc Cube protocol, the ganglion cell analysis measures GCIPLT segmentally in six wedge-shaped sectors (excluding a 1-mm-diameter region around the fovea) [28].

### Visual field testing

All patients underwent SAP testing using the Humphrey Field Analyzer 24-2 protocol (Carl Zeiss Meditec, Inc., Dublin, CA, USA). Visual fields with more than 20% fixation losses, MD < − 2 dB, false-negative errors, and false-positive errors were excluded. Using HFA SITA 24-2 results, only participants with visual fields corresponding to stage 0 (no visual field losses) following the Glaucoma Staging System (GSS 2) will be considered [29].

### RGC count estimation

Estimated RGC count was calculated with the CSFI in accordance with formulas derived by Meideros et al. [24, 30]. The first step involves estimating RGC count using SAP sensitivity (*s*) values in *d*B at a given eccentricity (ec). The following formulas were used to determine SAP-derived RGC count (SAPrgc):

1 $$\begin{gathered} m = \left[ {0.54\left( {{\text{ec}}*1.32} \right)} \right] + 0.9 \hfill \\ b\; = \;\;\left[ { - 1.5\;\left( {{\text{ec}} + 1.32} \right)} \right]\;--\;14.8 \hfill \\ gc = \;\frac{{\left( {s - 1} \right) - b}}{m} + 4.7 \hfill \\ {\text{SAPrgc}} = \;\sum {10^{\left( {{\text{gc}}*0.1} \right)}} \hfill \\ \end{gathered}$$

In the above formulas, m and b represent the slope and intercept, respectively, of a linear function that relates ganglion cell count (gc) to s at a given ec. All RGC densities were uniform within each individual test location corresponding to 6 × 6 degrees of visual space.

SD-OCT-derived RGC count (OCTrgc) was determined with the following formulas:

2 $$ \begin{gathered} d\; = \;\;\left( { - 0.007 + {\text{age}}} \right)\; + \;1.4 \hfill \\ c\; = \;\;\left( { - 0.26*{\text{MD}}} \right)\; + 0.12 \hfill \\ a = \;{\text{average\;RNFLT}}*10870*d \hfill \\ {\text{OCTrgc}} = \;{10^{\left\{ {\left[ {\log \left( a \right)*10 - c} \right]*0.1} \right\}}} \hfill \\ \end{gathered}$$

In the above formulas, d corresponds to axonal density (axons/um^2^) and c is a correction factor that considers the degree of functional visual impairment in order to account for retinal nerve fiber layer remodeling in advanced disease [24]. Estimated RGC count was obtained using the following formula:

3 $$ {\text{Estimated\;RGC\;count}} = \left( {1 + \frac{{{\text{MD}}}}{30}} \right)*{\text{OCTrgc + }}\left( { - \frac{{{\text{MD}}}}{30}} \right)*{\text{SAPrgc}} $$

Further rationale has been previously described in detail by Medeiros et al. [24].

### ssPERG testing

The ssPERG in this study follows the PERGLA protocol developed by Porciatti et al. [18], which was developed to simplify PERG-assisted glaucoma screening. The PERGLA protocol adds filters and amplifiers to ssPERG recordings to achieve an amplitude and signal-to-noise ratio adherent to the International Society for Clinical Electrophysiology of Vision (ISCEV) standards [8, 17, 31, 32].

The ssPERG was recorded using Diopsys^®^ NOVA-PERG (Diopsys, Inc., Pine Brook, New Jersey, USA) and was described previously [20]. Tests were performed in a dark room to standardized environment luminance, free of visual, and audible distractions. Subject’s forehead skin was cleaned using NuPrep^®^ Skin Prep Gel (Weaver and Company, CO, USA) and the lower eyelids using OCuSOFT^®^ Lid Scrub Original (OCuSOFT^®^ Inc., Rosenberg, TX, USA) to ensure good and stable electrical activity. Disposable hypoallergenic skin sensors Silver/Silver Chloride ink (Diopsys^®^ proprietary skin sensor) were applied on the lower lid of both eyes, at the lid margin and avoiding eyelashes. One ground sensor (Diopsys^®^ EEG electrode) was applied in the central forehead area with a small amount of conductive paste (Ten20^®^, Weaver and Company), and cables from the Diopsys NOVA device were connected to the electrodes. A total of three electrodes were used per test per patient (two active/reference and one ground electrodes, *Supplemental Fig. 1*). Subjects were fitted with the appropriate correction for a viewing distance of 24 inches and were instructed to fixate on a target at the center of the monitor in front of them [20].

The stimulus was presented on a gamma-corrected Acer V176L bm 17-inch monitor, having a refresh rate of 75 frames/s. Luminance output over time was verified using a luminance meter MavoSpot 2 USB (Gossen, GmbH, Nuremberg; Germany). The pattern stimulus consisted of black/white alternating square bars, reversing at 15 reversals/s (rps) with a duration of 25 s for high contrast [HC 85%] and 25 s for low contrast [LC 75%] for a total of 50 s per eye. The stimulus field subtends a visual angle of 1439.90 arc min. Each bar will subtend 22.49 arc min, for a total of 64 bars. A red target subtending 50.79 arc min was used as a fixation target and was centered on the stimulus field. The luminance of the white bars for 85% and 75% contrast was 204 cd/m^2^ and the luminance for black was 20.5 cd/m^2^ and 52.5 cd/m^2^, yielding a mean luminance of 112.3 cd/m^2^ and 128.2 cd/m^2^, respectively. All recorded signals underwent band filtration (0.5–100 Hz), amplification (gain = 20,000), and averaging at least 150 frames. The signal was sampled at 1920 samples per second by an analog-to-digital (A/D) converter. The voltage range of the (A/D) converter was programmed between − 5 V and + 5 V. Sweeps contaminated by eye blinks or gross eye saccades were automatically rejected if they exceeded a threshold voltage of 50 μV, and these sections were identified as artifacts in the report. Synchronized single-channel electroretinography was recorded, generating a time series of 384 data points per analysis frame (200 ms). An automatic fast Fourier transformation was applied to the PERG waveforms to isolate the desired component at 15 rps. Other frequencies, such as those originating from eye muscles, were rejected. The PERG test results were saved in a SQL database and presented in a report form to be used for analysis [20]. For every subject, four pre-programmed full “contrast sensitivity protocols” were performed sequentially, which consisted of two 25 s recordings for each eye: first with high-contrast (85%) diffuse retinal stimulation, and then with low-contrast (75%) pattern stimulation. The device collected 5 frames of data per second, totaling 125 frames of data, and the first 10 frames (2 s) of data were discarded [20].

For each eye, three PERG parameters (Magnitude [Mag], MagnitudeD [MagD], and MagD/Mag ratio) were measured. Mag (µV) represents the amplitude of the signal strength at the specific reversal rate of 15 Hz in the frequency domain, while MagD (µV) represents an adjusted amplitude of the PERG signal impacted by phase variability throughout the waveform recording. MagnitudeD is considered to be equal to the Mag, which was altered by phase change, and therefore it is also considered to reflect phase consistency [20].

A recording where the phase of the response is consistent will produce a MagD value close to that of the Mag, whereas a recording where the phase of the response varies will produce a MagD value lower than that of Mag. Averaging responses that are out of phase with each other will cause some degree of cancellation. The MagD/Mag ratio is a ratio that is a within-subject representation of the phase consistency of ssPERG. Due to the inability to normalize MagD/Mag ratio in our cohort, values for this parameter were not reported.

These parameters are repeatable, reproducible, and sufficiently reliable in clinical practice [17]. Results were also presented in a color-coded system, like “traffic light system,” with green color showing the results being within reference range, yellow color representing values within borderline reference range, and red color representing results outside reference range.

### Statistical analysis

Shapiro–Wilk test was used to determine the normality of the distribution for all important variables. Descriptive statistics were used to evaluate continuous and demographic data. Mean and standard deviation values were determined for Mag and MagD, HFA SITA Standard (24-2) tests, and all SD-OCT variables. Group differences between healthy controls and GS were analyzed using independent sample *t* tests for continuous variables and chi-square tests for categorical variables. Associations among continuous variables were analyzed using Pearson correlations. ssPERG parameters (Mag and MagD) achieved normal distribution through log10 transformations (*Shapiro–Wilk, p* ≥ *0.38).* All normalized data were within ± 3 standard deviations of the mean.

### Mediation analyses

A total of six mediation analyses were conducted on ssPERG parameters using SPSS PROCESS v3.5 by Andrew Hayes [33]. Each mediation analysis describes the effect of an independent variable (*X*) on a dependent variable (*Y*) through a mediation variable (*M*). We performed mediation analyses with ssPERG parameters (Mag and MagD) as *X*, estimated RGC count as *M*, and ARNFLT, MD, and average GCIPLT as *Y*, for a total of six analyses. For each analysis, the direct effect of *X* on *Y* is the correlation coefficient from a linear regression of *X* on *Y*. The indirect effect of *X* on *Y* is the product of the correlation coefficients of *X* on *M* and *M* on *Y*. The significance of the indirect and direct effects of *X* on *Y* was determined using 5000 bootstrapped samples with a 95% confidence interval (CI). The strengths and limitations of this mediation analysis have been previously described [34].

## Results

**Fig. 1:**
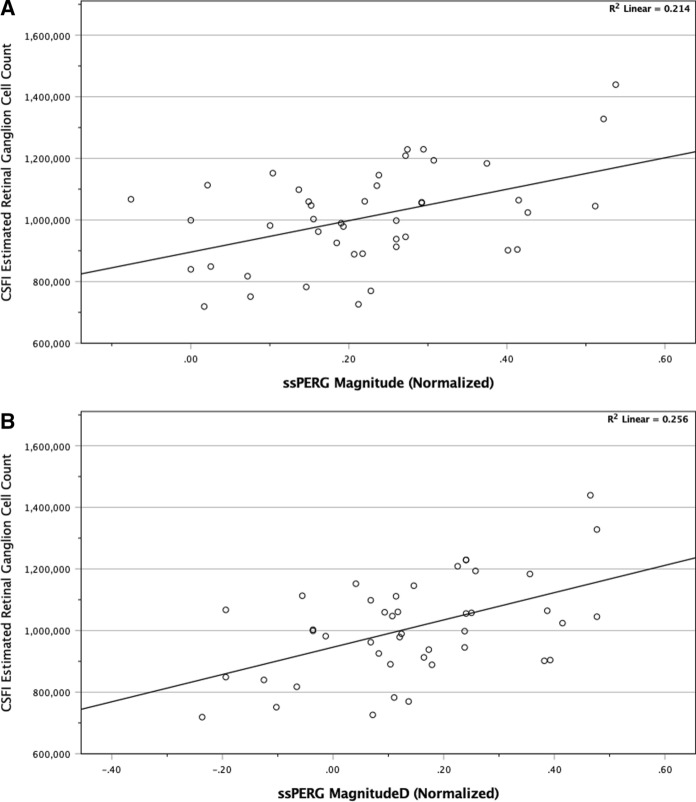
Scatterplot diagrams showing the associations among estimated RGC count with **A** normalized Mag and **B** normalized MagD

Independent sample *t* tests revealed a significantly reduced ARNFLT, GCIPLT, estimated RGC count, Mag, and MagD in GS relative to healthy subjects *(p* ≤ *0.001 for all parameters)* (Table 1). Pearson correlations revealed significant linear associations among both ssPERG parameters and ARNFLT *(r* ≥ *0.53, p* < *0.001)*, GCIPLT *(r* ≥ *0.38, p* ≤ *0.011),* and estimated RGC count *(r* ≥ *0.46, p* ≤ *0.002)* (Table 2). Figure 1 shows a scatterplot demonstrating the associations between ssPERG parameters and estimated RGC count.

**Table 1:** Summary of demographic characteristics of healthy subjects and GS

	Healthy subjects (*n* = 30 eyes)	Glaucoma suspects (*n* = 14 eyes)		
	Mean (± SD)	Mean (± SD)	*p* value	Cohen’s d effect size
Age (years)	49 ± 12	56 ± 19	0.22	− 0.486
Female (%)	33	50	0.32	− 0.339
Intraocular pressure (mmHg)	17.1 ± 3.9	16.8 ± 3.8	0.78	0.318
24-2 MD (dB)	0.1 ± 1.1	− 0.2 ± 1.0	0.31	1.209
ARNFLT (μM)*	96.80 ± 7.95	82.86 ± 5.95	< 0.001	2.384
GCIPLT (μM)*	81.27 ± 5.95	75.29 ± 4.50	0.001	2.303
Mag (μV)*	1.95 ± 0.64	1.36 ± 0.31	< 0.001	1.919
MagD (μV)*	1.72 ± 0.64	1.06 ± 0.33	< 0.001	2.621
Normalized Mag*	0.27 ± 0.14	0.12 ± 0.10	< 0.001	2.096
Normalized MagD*	0.21 ± 0.16	0.00 ± 0.15	< 0.001	2.822
Estimated RGC count*	1.07e6 ± 1.27e5	8.64e5 ± 1.19e5	< 0.001	2.755

*RGC* retinal ganglion cell, *SD* standard deviation, *MD* mean deviation, *ARNFLT* average retinal nerve fiber layer thickness, *GCIPLT* average ganglion cell layer and inner plexiform layer thickness, *Mag* magnitude, *MagD* magnitude, *RGC* retinal ganglion cell.

*p ≤ 0.001.

**Table 2:** Summary of correlations between ssPERG parameters, CSFI estimated RGC count, and measures of structure and function

	*r*	*p* value
*Normalized Mag*		
24-2 MD (dB)	0.22	0.15
ARNFLT (μM)**	0.53	< 0.001
GCIPLT (μM)*	0.38	0.011
Estimated RGC count*	0.46	0.002
*Normalized MagD*		
24-2 MD (dB)	0.23	0.13
ARNFLT (μM)**	0.56	< 0.001
GCIPLT (μM)*	0.40	0.007
Estimated RGC count**	0.51	< 0.001

All relationships are expressed as Pearson and partial correlations adjusted for age. ssPERG parameters Mag, MagD, and MagD/Mag ratio are expressed as transformed data. *ssPERG* steady-state pattern electroretinography, *Mag* magnitude, *MagD* magnitude, *MD* mean deviation, *ARNFLT* average retinal nerve fiber layer thickness, *GCIPLT* ganglion cell layer and inner plexiform layer thickness.

*p < 0.05, **p < 0.001.

**Fig. 2:**
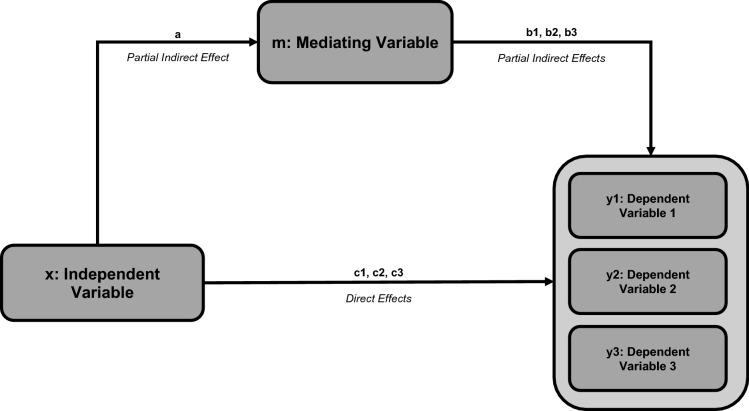
This schematic summarizes the results of three mediation analyses conducted for an independent variable (*x*) through a mediating variable (*m*) on three dependent variables (*y*1, *y*2, and *y*3). “*a*” represents the partial indirect effect of *x* on *m*. As “*a*” depends on *x* and *m*, but not *y*, it is constant in all three mediation analyses. “*b*1,” “*b*2,” and “*b*3” represent the partial indirect effects of *m* on *y*1, *y*2, and *y*3, respectively. Indirect effects for *y*1, *y*2, and *y*3 are calculated by multiplying “*a*” by “*b*1,” “*b*2,” and “*b*3,” respectively. “*c*1,” “*c*2,” and “*c*3” represent the direct effects of *x* on *y*1, *y*2, and *y*3, respectively

**Fig. 3:**
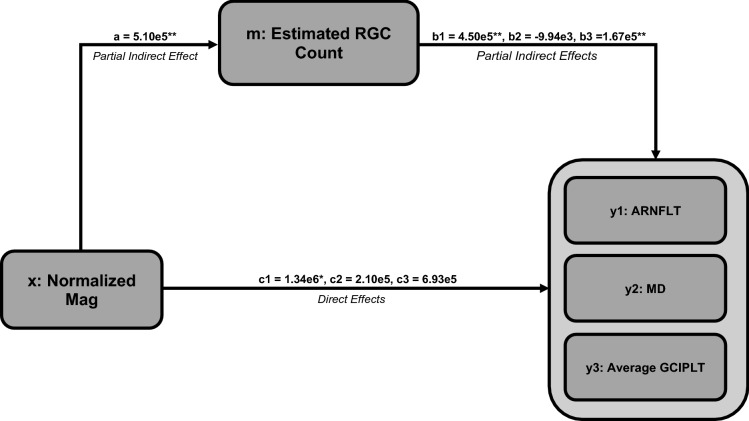
Summary of three mediation analyses with normalized Mag as the independent variable (*x*), estimated RGC count as the mediating variable (*m*), and ARNFLT (*y*1), MD (*y*2), and average GCIPLT (*y*3) as the dependent variables. “*a*” is the partial indirect effect of *x* on m. “*b*1,” “*b*2,” and “*b*3” are the partial indirect effects of *m* on *y*1, *y*2, and *y*3, respectively. “*c*1,” “*c*2,” and “*c*3” are the direct effects of *x* on *y*1, *y*2, and *y*3, respectively. Mag, magnitude (μV); RGC, retinal ganglion cell; ARNFLT, average retinal nerve fiber layer thickness (μM); MD, mean deviation (dB); GCIPLT, ganglion cell layer and inner plexiform layer thickness (μM). *p < 0.05, **p < 0.01

**Fig. 4:**
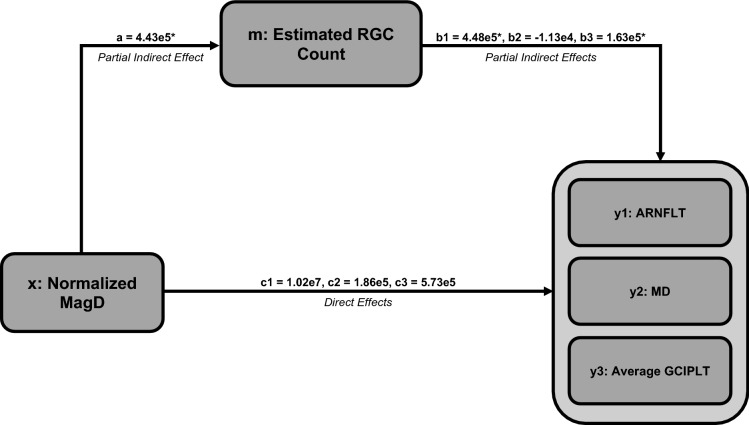
Summary of three mediation analyses with normalized MagD as the independent variable (*x*), estimated RGC count as the mediating variable (*m*), and ARNFLT (*y*1), MD (*y*2), and average GCIPLT (*y*3) as the dependent variables. “*a*” is the partial indirect effect of *x* on m. “*b*1,” “*b*2,” and “*b*3” are the partial indirect effects of *m* on *y*1, *y*2, and *y*3, respectively. “*c*1,” “*c*2,” and “*c*3” are the direct effects of *x* on *y*1, *y*2, and *y*3. MagD, transformed magnitudeD; RGC, retinal ganglion cell; ARNFLT, average retinal nerve fiber layer thickness (μM); MD, mean deviation (dB); GCIPLT, ganglion cell layer and inner plexiform layer thickness (μM). *p < 0.01

To explain the relationships among ssPERG parameters, estimated RGC count, and measures of structure and function, six mediation analyses were performed with ssPERG parameters as independent variables (X), estimated RGC count as the mediating variable (M), and ARNFLT, 24-2 MD, and GCIPLT as dependent variables (Y) (Fig. 2). The indirect effects of Mag on ARNFLT *(2.30e6, 95% CI [7.01e5, 4.05e6])* and GCIPLT (*8.50e5, 95% CI [1.60e5, 1.87e6]*) were significant. The direct effect of Mag on ARNFLT (*1.34e6, 95% CI [1.47e4, 2.66e6]*) was significant (Fig. 3). The indirect effects of MagD on ARNFLT (*1.92e6, 95% CI [7.97e5, 3.28e6]*) and GCIPLT (*7.20e5, 95% CI [1.65e5, 1.52e6]*) were significant, whereas the direct effects of MagD on ARNFLT and GCIPLT were not significant (*Fig. **4*). The indirect and direct effects of both Mag and MagD on MD were not significant.

**Fig. 5:**
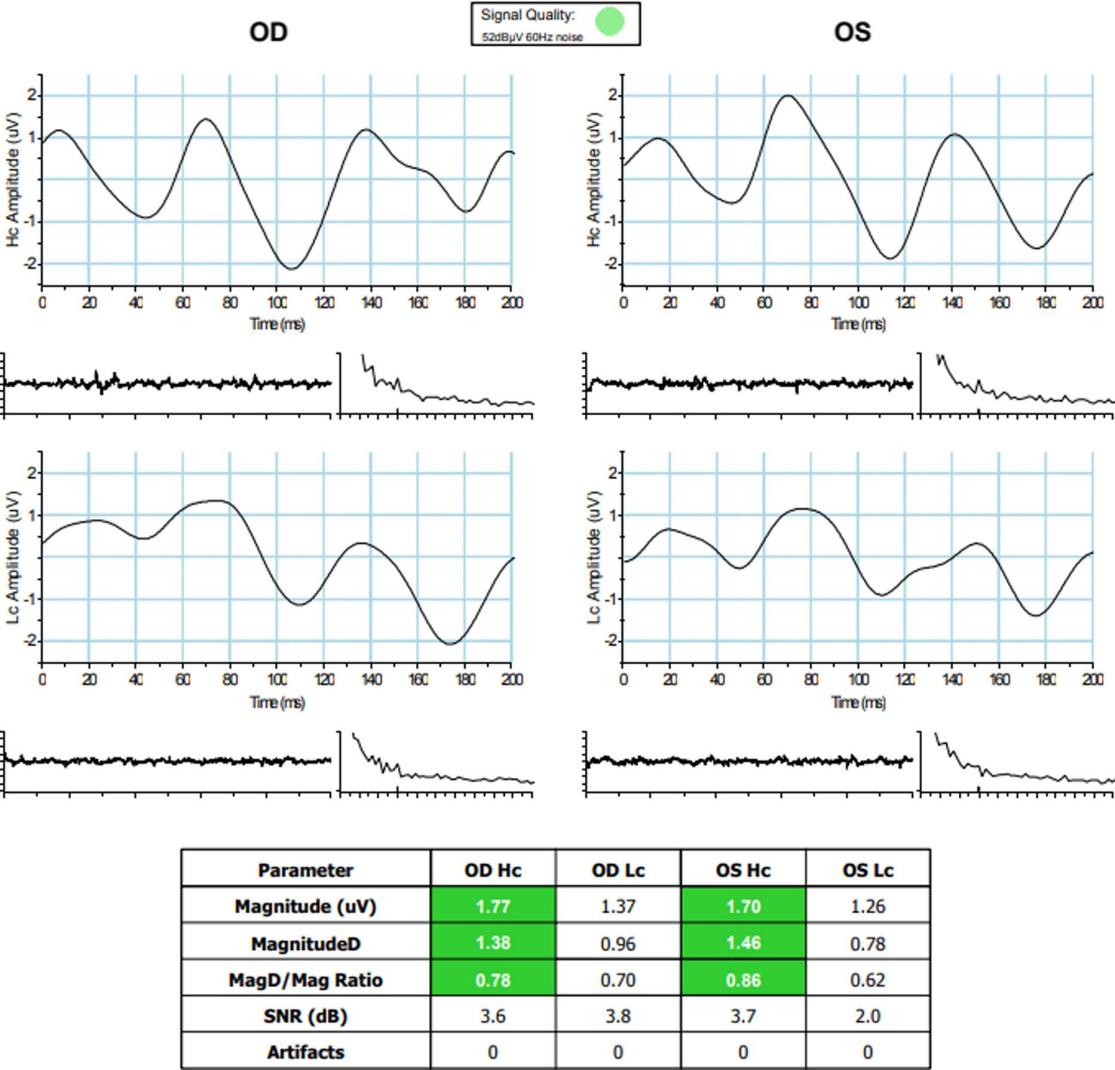
Example of a healthy subject with normal ssPERG results. ssPERG, steady-state pattern electroretinography; Mag, magnitude (μV); MagD, magnitudeD (μV); Hc, high-contrast; Lc, low-contrast; SNR, signal-to-noise ratio (dB)

**Fig. 6:**
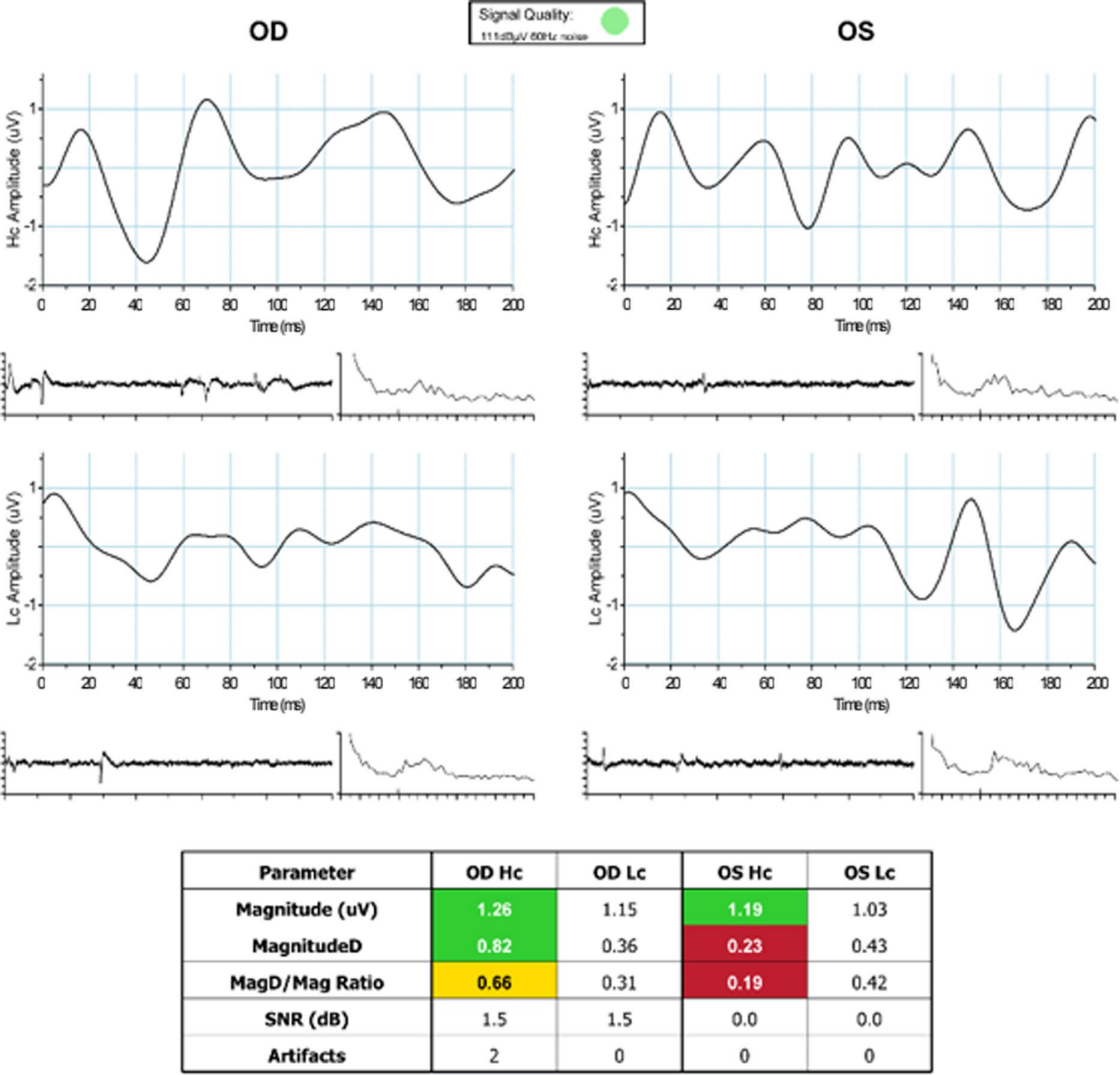
Example of a GS subject with abnormal ssPERG results. GS, glaucoma suspects; ssPERG, steady-state pattern electroretinography; Mag, magnitude (μV); MagD, magnitudeD (μV); Hc, high-contrast; Lc, low-contrast SNR, signal-to-noise ratio (dB)

**Fig. 7:**
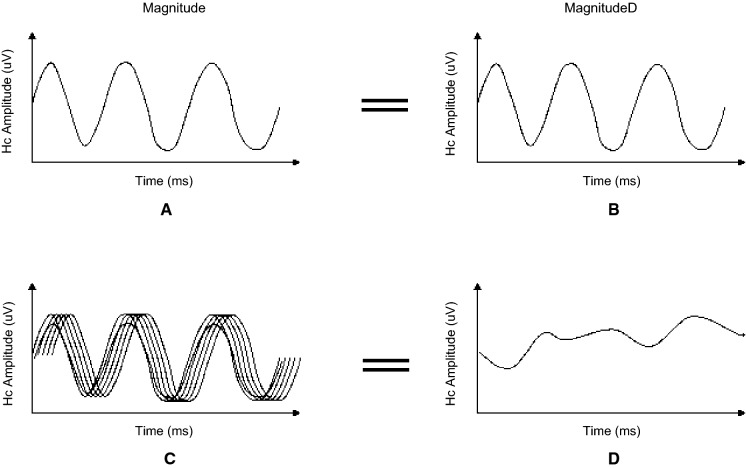
Schematic diagram demonstrating the effect of phase variability on ssPERG MagD. **A** Mag waveforms of a normal RGC response in ssPERG testing without phase variability. RGCs respond to multiple cycles of stimulus uniformly. **B** MagD waveforms without phase variability are identical to Mag waveforms. **C** Mag waveforms in ssPERG testing with phase variability. RGCs exhibit delayed responses to multiple cycles of stimuli. **D** MagD waveforms are dissimilar to Mag waveforms due to signal cancellation. ssPERG, steady-state pattern electroretinography; Mag, magnitude (μV); MagD, magnitudeD (μV); Hc, high contrast; RGC, retinal ganglion cell

Examples of ssPERG waveforms in healthy and GS subjects are shown in Figs. 5 and 6, respectively. Figure 7 is a diagram to show the effect of phase on MagD. In an ideal subject without phase changes (Fig. 7A), multiple cycles of stimulus yield uniform RGC cell responses. The resultant Mag waveforms “stack” upon each other and appear as a singular waveform (Fig. 7B). Without phase variability, there is no degree of cancellation between multiple Mag waveforms, and MagD is equal to Mag (Fig. 7A, B). In a subject with phase changes, RGCs exhibit variable responses to multiple cycles of stimulus. The differences in Mag at a given time interval exhibit a degree of signal cancellation, yielding a MagD that is less than Mag (Fig. 7C, D).

## Discussion

In this study, we investigated whether estimated RGC count was lower in GS eyes relative to healthy eyes and sought to determine whether estimated RGC count mediated the associations between ssPERG and SD-OCT measures. As estimated RGC count was shown capable of predicting the rate of glaucoma progression [27], such findings would suggest that ssPERG has the potential to identify GS subjects at the highest risk of developing early glaucoma.

The average estimated RGC count in healthy (1.07e6 ± 1.27e5, Table 1) and GS (8.64e5 ± 1.19e5) eyes in this study were consistent with prior reports that estimated RGC count in similar study groups. For example, Tatham et al. evaluated the relationship between estimated RGC count and cup-to-disc ratio and reported an average estimated RGC count of 1.06e6 ± 1.74e5 in healthy eyes and 8.29e5 ± 1.37e5 in GS [35]. As expected, both Mag and MagD significantly correlated with ARNFLT (*r* ≥ 0.53, *p* < 0.001) and GCIPLT (*r* ≥ 0.38, *p* ≤ 0.011) (Table 2), which are also consistent with prior reports [12]. For example, Bowd et al. [12] associated PERG amplitude with SD-OCT measures and reported correlations with ARNFLT (*r* = 0.42, *p* < 0.001) and ganglion cell complex thickness (*r* = 0.42, *p* < 0.001).

The most important results of our study were the linear associations among ssPERG parameters (Mag, MagD) and estimated RGC count (Fig. 1) and the results of our mediation analyses. We found that estimated RGC count fully mediated the relationship between ssPERG parameters and GCIPLT. Estimated RGC count also fully mediated the relationship between MagD and ARNFLT, but only partially mediated the relationship between Mag and ARNFLT (Figs. 3, 4). This implies that the relationship between RGC dysfunction and RNFL thinning in GS was fully explained by RGC losses only when phase latency (MagD) was considered (Figs. 3, 4).

The relevance of these results relies on two key hypotheses supported by previous reports investigating the pathophysiology of ssPERG losses in glaucoma. The first hypothesis is that ssPERG amplitude and phase latency measure unrelated aspects of RGC function, with amplitude measuring the electrical activity generated by RGCs in response to a light stimulus and phase latency measuring the temporal dynamics of RGC neural pathways [36]. The second is that the temporal dynamics of RGC pathways are dependent on the morphological integrity of RGC axons, dendritic trees, and synapses [36–40].

To support these hypotheses, consider a series of experiments conducted by Porciatti et al. that simulated diminished RGC function and diminished RGC count in healthy subjects to better understand the pathophysiology of PERG amplitude and phase [36]. To simulate diminished RGC function, both luminance (the overall light intensity of the stimulus) and contrast (the difference in luminance between the dark and light boxes of the stimulus at a constant mean luminescence) were reduced. Amplitude losses occurred from both reduced luminance and contrast, but phase latency increased with reduced luminance only. To simulate diminished RGC count, the area of the PERG stimulus was reduced, which also decreased amplitude without increasing phase latency [36]. These findings suggested that diminished overall activity of RGCs, whether it be due to death or dysfunction, could explain amplitude losses is glaucoma but not phase latency. To explain phase latency, one must consider factors that reduce the luminal input to RGCs and are independent of RGC count.

Therefore, the most likely factors to explain phase latency in glaucoma are characteristic changes in RGC morphology that reduce their functional capacity [36], as RGC dendritic tree atrophy, axonal degeneration, and cell body shrinkage have all been described in the literature to contribute to RGC dysfunction in early glaucoma that precedes RGC death [37, 39, 40]. For example, Weber et al. stained RGCs with fluorescent dye in primates to examine the degenerative effects of prolonged elevation of IOP on RGC morphology and found that elevated IOP caused dendritic tree atrophy and cell soma shrinkage before RGC death occurred [39]. Importantly, a follow-up study found that RGCs with reduced soma size and less dendritic arbor complexity exhibited normal electrical properties but were less spatially and temporally responsive to light stimulation [41], implying that changes in RGC morphology would increase ssPERG phase latency without affecting ssPERG amplitude. Furthermore, a mouse model of glaucoma provided evidence that RGC dendritic atrophy occurred before axonal atrophy, implying that loss of dendritic tree complexity was the first morphological change of RGCs exhibited in glaucomatous eyes [40]. Since RGC electrophysiological function is dependent on RGC morphological integrity [38, 39], it is reasonable to suspect that ssPERG may be capable of detecting these changes before cell death occurs. In this respect, at least two studies demonstrated greater than a 50% decrease in PERG signal within one day following an optic nerve crush in mice despite an absence of RGC death during this timeframe [42, 43], and our previous study demonstrated that ssPERG correlated with morphological changes of the optic nerve head in early glaucoma [20].

The current study utilized Mag and MagD to quantify amplitude and phase latency, respectively. Mag is the amplitude of signal strength at a specific reversal rate, and MagD is a measure of Mag adjusted for phase latency (rather than a direct measurement of phase) (see Methods, Fig. 7). As expected, we found that normalized MagD was similar to Mag in healthy eyes (*0.21* ± *0.16 vs. 0.27* ± *0.14, respectively*), but lower than Mag in GS eyes (*0.00* ± *0.15 vs. 0.12* ± *0.10, respectively*) (Table 1), which agreed with prior reports of increased phase latency in GS eyes [44]. Our mediation analyses demonstrated that estimated RGC count fully mediated the relationship between ssPERG parameters (Mag and MagD) and GCIPLT, which implies that the relationship between RGC functional losses and GCIPLT losses was fully explained by RGC losses (Figs. 3, 4). However, we also found that estimated RGC count only partially mediated the relationship between Mag and ARNFLT, which implies that the relationship between RGC functional losses and RNFL thinning was *not* fully explained by the number of RGCs lost (Fig. 3). Indeed, estimated RGC count only fully mediated the relationship between MagD and ARNFLT, which suggests that *phase latency also impacted the relationship between RGC function and ARNFLT* (Fig. 4).

In this study, it is important to recall that increased phase latency in GS is likely associated with changes in RGC morphology, namely dendritic arbor atrophy, and these changes were shown to precede axon atrophy [37, 39, 40]. From an anatomical perspective, this would imply that GCIPL thinning would precede some degree of RNFL thinning, as RGC dendritic arbors and cell bodies are located in the GCIPL and RGC axons are located in the RNFL [45]. If such changes are the generators of phase latency, then it is reasonable to infer that eyes exhibiting a higher degree of phase latency also have a higher proportion of morphologically compromised RGCs. In this respect, our results suggest that dendritic atrophy and cell soma shrinkage played a significant role in RNFL thinning in our study subjects. In other words, RGC dysfunction resulted in RNFL thinning in our study subjects due to both RGC death and changes in RGC morphology.

While this study provides evidence that ssPERG parameters and estimated RGC count are associated, we do not suggest that ssPERG parameters are related to “true” RGC count. Raza and Hood [46] demonstrated that the Harwerth model for estimating RGC count, which is the foundation for the CSFI, overpredicted RGC count at all degrees of retinal eccentricity relative to histological RGC count in postmortem humans [47], and thus, RGC estimates may lack accuracy in human subjects. We therefore intended for estimated RGC counts to be interpreted relative to each other and not as empiric values. Nonetheless, to the best of our knowledge this is the first study to establish a linear relationship between functional testing measures and the CSFI. As ssPERG measures RGC function, these linear relationships support the assumption that the CSFI is related to RGC count, regardless of whether the numerical values of RGC estimates are quantitatively accurate.

The present study has limitations. Although age was not significantly different between groups, there was a wide age range in our selection criteria. As age is a risk factor for RGC loss that is independent of glaucoma, the wide age range may have contributed to large standard deviations of estimated RGC count. Furthermore, RGC counts were estimated utilizing an algorithm derived from primates [46], and histological verification of these estimates is not possible. However, estimated RGC counts closely resembled histologically verified counts in human’s postmortem in some studies [4, 26]. Our data analysis was also limited by Cirrus SD-OCT, which does not measure ganglion cell layer and inner plexiform layer thicknesses independent of each other. Future investigations should consider using other OCT devices such as Spectralis SD-OCT, as ganglion cell layer thickness and inner plexiform layer thickness can be measured separately. One major limitation was the inability to determine directionality from mediation analyses due to the cross-sectional nature of this study. However, mediation analyses can be useful in cross-sectional studies when directionality has been predetermined [33]. Prior investigations showed that ssPERG losses preceded structural losses in glaucoma [6, 15, 20, 47, 48], in some studies by several years, and to the best of our knowledge, there have been no reports that showed ARNFLT or GCIPLT losses in GS preceding ssPERG losses. From a biological plausibility perspective, RGC dysfunction can be assumed to occur before RGC death [49], and recent investigations demonstrated that ssPERG is capable of detecting RGC dysfunction before death occurs in glaucoma subjects [5, 6, 50, 51]. It was also shown that RGC death preceded ARNFLT and ganglion cell complex losses in animal models [52, 53], and investigations of directionality between these two variables are not possible in living humans. Taken together, we feel these data are sufficient to suggest that ssPERG losses occur before RGC death, and RGC death in turn results in ARNFLT and GCIPLT losses. Our mediation analyses provided evidence to support this notion, and longitudinal studies are warranted to confirm the directionality between these variables in future investigations.

## Conclusion

The results presented in this study provide evidence that the CSFI and ssPERG parameters are significantly associated. ssPERG correlated with estimated RGC count, and the relationships among ssPERG losses and SD-OCT losses in GS were mediated by RGC losses. Estimated RGC count fully mediated the relationship between ssPERG parameters and ARNFLT only after phase latency was considered, implying that temporal dysfunction of the RGC neural pathway contributes to RNFL thinning. Taken together, these findings suggest that ssPERG parameters hold potential for identifying GS at high risk of glaucoma progression.

## Supplementary Information

Below is the link to the electronic supplementary material.

Supplementary file1 (DOCX 45 KB)

Supplementary file2 (DOCX 25 KB)

## Declarations

### Statement on the welfare of animals

This study was not conducted on animals.
