# Supplementary figures and images for: Associations between steady-state pattern electroretinography and estimated retinal ganglion cell count in glaucoma suspects
Source: Doc Ophthalmol. 2022 Apr 4;145(1):11–25. doi: 10.1007/s10633-022-09869-9 (PMC9259521; doi:10.1007/s10633-022-09869-9)

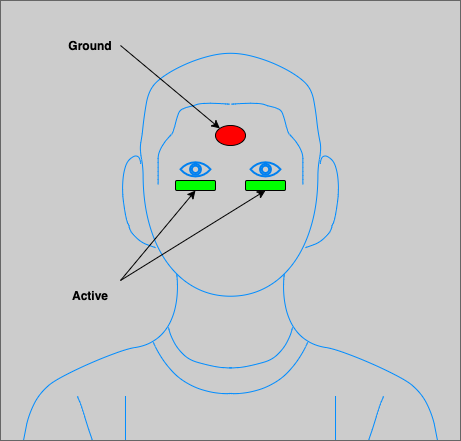


**Supplemental Figure 1.** Schematic representation of electrode placement in our study subjects.

Supplement: Supplementary file 1 — Supplementary file1 (DOCX 45 KB) [file 10633_2022_9869_MOESM1_ESM.docx]
