# Supplementary material for: Associations between steady-state pattern electroretinography and estimated retinal ganglion cell count in glaucoma suspects
Source: Doc Ophthalmol. 2022 Apr 4;145(1):11–25. doi: 10.1007/s10633-022-09869-9 (PMC9259521; doi:10.1007/s10633-022-09869-9)
Supplement: Supplementary file 2 — Supplementary file2 (DOCX 25 KB) [file 10633_2022_9869_MOESM2_ESM.docx]

**Supplemental Table 1.** Mediation Analysis of Mag (X), Estimated RGC Count/100,000 (M), and ARNFLT (Y).

| **Model Summary** | **R** | **MSE** | **F** | **df1** | **df2** | **p** |
| --- | --- | --- | --- | --- | --- | --- |
| X on M | 0.46 | 2.03 | 11.47 | 1.00 | 42.00 | < 0.001 |
| X on Y | 0.53 | 70.61 | 16.74 | 1.00 | 42.00 | < 0.001 |
|  |  |  |  |  |  |  |
| **Model: X on M** | **Coefficient** | **SE** | **t** | **LLCI** | **ULCI** | **p** |
| Constant | 8.96 | 0.40 | 22.56 | 8.16 | 9.76 | < 0.001 |
| Normalized Mag | 5.10 | 1.51 | 3.39 | 2.0603 | 8.1386 | < 0.001 |
|  |  |  |  |  |  |  |
| **Model: X on Y** | **Coefficient** | **SE** | **t** | **LLCI** | **ULCI** | **p** |
| Constant | 43.98 | 5.54 | 7.94 | 79.59 | 89.03 | < 0.001 |
| Normalized Mag | 13.36 | 6.54 | 2.04 | 0.15 | 26.58 | 0.047 |
| Estimated RGC Count | 4.50 | 0.59 | 7.58 | 3.30 | 5.70 | < 0.001 |
|  |  |  |  |  |  |  |
| **Effects of X on Y** | **Effect** | **SE** | **LLCI** | **ULCI** | **t** | **p** |
| Total Effect | 36.32 | 8.88 | 18.41 | 54.24 | 4.09 | < 0.001 |
| Direct Effect | 13.36 | 6.54 | 0.15 | 26.58 | 2.04 | 0.047 |
| Indirect Effect | 22.96 | 8.39 | 7.68 | 40.37 |  |  |

**Supplemental Table 2.** Mediation Analysis of Mag (X), Estimated RGC Count/100,000 (M), and GCIPLT (Y).

| **Model Summary** | **R** | **MSE** | **F** | **df1** | **df2** | **p** |
| --- | --- | --- | --- | --- | --- | --- |
| X on M | 0.46 | 2.03 | 7.13 | 1.00 | 42.00 | 0.002 |
| X on Y | 0.55 | 24.88 | 9.05 | 2.00 | 41.00 | < 0.001 |
|  |  |  |  |  |  |  |
| **Model: X on M** | **Coefficient** | **SE** | **t** | **LLCI** | **ULCI** | **p** |
| Constant | 8.96 | 0.40 | 22.56 | 8.16 | 9.76 | < 0.001 |
| Normalized Mag | 5.10 | 1.51 | 3.39 | 2.06 | 8.16 | 0.002 |
|  |  |  |  |  |  |  |
| **Model: X on Y** | **Coefficient** | **SE** | **t** | **LLCI** | **ULCI** | **p** |
| Constant | 61.02 | 5.03 | 12.12 | 50.85 | 71.18 | < 0.001 |
| Normalized Mag | 6.93 | 5.94 | 1.17 | - 5.07 | 18.94 | 0.25 |
| Estimated RGC Count | 1.67 | 0.54 | 3.09 | 0.58 | 2.76 | 0.004 |
|  |  |  |  |  |  |  |
| **Effects of X on Y** | **Effect** | **SE** | **LLCI** | **ULCI** | **t** | **p** |
| Total Effect | 15.43 | 5.78 | 3.77 | 22.11 | 2.67 | 0.011 |
| Direct Effect | 6.93 | 5.94 | - 5.07 | 18.94 | 1.17 | 0.25 |
| Indirect Effect | 8.50 | 4.46 | 1.60 | 19.25 |  |  |

**Supplemental Table 3.** Mediation Analysis of MagD (X), Estimated RGC Count/100,000 (M), and ARNFLT (Y).

| **Model Summary** | **R** | **MSE** | **F** | **df1** | **df2** | **p** |
| --- | --- | --- | --- | --- | --- | --- |
| X on M | 0.51 | 1.92 | 14.47 | 1.00 | 42.00 | 0.005 |
| X on Y | 0.84 | 30.55 | 47.39 | 2.00 | 41.00 | < 0.001 |
|  |  |  |  |  |  |  |
| **Model: X on M** | **Coefficient** | **SE** | **t** | **LLCI** | **ULCI** | **p** |
| Constant | 9.46 | 0.27 | 35.49 | 8.92 | 10.00 | < 0.001 |
| Normalized MagD | 4.43 | 1.16 | 3.80 | 2.08 | 6.78 | < 0.001 |
|  |  |  |  |  |  |  |
| **Model: X on Y** | **Coefficient** | **SE** | **t** | **LLCI** | **ULCI** | **p** |
| Constant | 45.76 | 5.91 | 7.74 | 33.81 | 57.70 | < 0.001 |
| Normalized MagD | 10.16 | 5.38 | 1.89 | - 0.70 | 21.02 | 0.07 |
| Estimated RGC Count | 4.48 | 0.61 | 7.28 | 3.23 | 5.72 | < 0.001 |
|  |  |  |  |  |  |  |
| **Effects of X on Y** | **Effect** | **SE** | **LLCI** | **ULCI** | **t** | **p** |
| Total Effect | 29.98 | 6.94 | 15.97 | 22.11 | 4.32 | 0.001 |
| Direct Effect | 10.16 | 5.38 | - 0.70 | 21.02 | 1.89 | 0.07 |
| Indirect Effect | 19.82 | 6.42 | 1.61 | 8.12 |  |  |

**Supplemental Table 4.** Mediation Analysis of MagD (X), Estimated RGC Count/100,000 (M), and GCIPLT (Y).

| **Model Summary** | **R** | **MSE** | **F** | **df1** | **df2** | **p** |
| --- | --- | --- | --- | --- | --- | --- |
| X on M | 0.51 | 1.92 | 14.48 | 1.00 | 42.00 | 0.005 |
| X on Y | 0.55 | 24.85 | 9.07 | 2.00 | 41.00 | 0.005 |
|  |  |  |  |  |  |  |
| **Model: X on M** | **Coefficient** | **SE** | **t** | **LLCI** | **ULCI** | **p** |
| Constant | 9.46 | 0.27 | 35.49 | 8.92 | 10.00 | < 0.001 |
| Normalized MagD | 4.43 | 1.16 | 3.80 | 2.08 | 6.78 | < 0.001 |
|  |  |  |  |  |  |  |
| **Model: X on Y** | **Coefficient** | **SE** | **t** | **LLCI** | **ULCI** | **p** |
| Constant | 62.14 | 5.33 | 11.65 | 51.37 | 72.91 | < 0.001 |
| Normalized MagD | 5.73 | 4.85 | 1.18 | -4.07 | 15.53 | 0.24 |
| Estimated RGC Count | 1.62 | 0.55 | 2.93 | 0.51 | 2.75 | 0.006 |
|  |  |  |  |  |  |  |
| **Effects of X on Y** | **Effect** | **SE** | **LLCI** | **ULCI** | **t** | **p** |
| Total Effect | 12.93 | 4.55 | 3.76 | 22.11 | 2.84 | 0.007 |
| Direct Effect | 5.73 | 4.85 | -4.07 | 15.53 | 1.18 | 0.24 |
| Indirect Effect | 7.20 | 3.51 | 1.61 | 15.28 |  |  |
